# TNF-α–mediated reduction in inhibitory neurotransmission precedes sporadic Alzheimer’s disease pathology in young *Trem2*^*R47H*^ rats

**DOI:** 10.1074/jbc.RA120.016395

**Published:** 2020-11-21

**Authors:** Siqiang Ren, Lionel Breuillaud, Wen Yao, Tao Yin, Kelly A. Norris, Simone P. Zehntner, Luciano D’Adamio

**Affiliations:** 1 Department of Pharmacology, Physiology & Neuroscience New Jersey Medical School, Brain Health Institute, Jacqueline Krieger Klein Center in Alzheimer's Disease and Neurodegeneration Research, Rutgers, The State University of New Jersey, Newark, New Jersey, USA; 2 Biospective Inc., Montreal, Quebec, Canada

**Keywords:** neurodegenerative disease, synaptic plasticity, rats, Alzheimer's disease, GABA, animal model, Trem2, Aβ, TNF-α, AD, Alzheimer’s disease, CNS, central nervous system, CSF, cerebrospinal fluid, FAD, familial Alzheimer’s disease, GABA, γ-aminobutyric acid, Ig, immunoglobulin, IHC, mmunohistochemistry, KI, knock-in, LTP, long-term potentiation, mIPSCs, miniature inhibitory postsynaptic currents, PPF, paired-pulse facilitation, SAD, sporadic Alzheimer’s disease, TNFIs, TNF-α–specific inhibitors, TREM2, triggering receptor expressed on myeloid cells 2

## Abstract

Alzheimer’s disease (AD) is a neurodegenerative dementia associated with deposition of amyloid plaques and neurofibrillary tangles, formed by amyloid β (Aβ) peptides and phosphor-tau, respectively, in the central nervous system. Approximately 2% of AD cases are due to familial AD (FAD); ∼98% of cases are sporadic AD (SAD). Animal models with FAD are commonly used to study SAD pathogenesis. Because mechanisms leading to FAD and SAD may be distinct, to study SAD pathogenesis, we generated *Trem2*^*R47H*^ knock-in rats, which carry the SAD risk factor p.R47H variant of the microglia gene triggering receptor expressed on myeloid cells 2 (*TREM2*). *Trem2*^*R47H*^ rats produce human-Aβ from a humanized-*App* rat allele because human-Aβ is more toxic than rodent-Aβ and the pathogenic role of the p.R47H *TREM2* variant has been linked to human-Aβ–clearing deficits. Using periadolescent *Trem2*^*R47H*^ rats, we previously demonstrated that supraphysiological tumor necrosis factor-α (TNF-α) boosts glutamatergic transmission, which is excitatory, and suppresses long-term potentiation, a surrogate of learning and memory. Here, we tested the effect of the p.R47H variant on the inhibitory neurotransmitter γ-aminobutyric acid. We report that GABAergic transmission is decreased in *Trem2*^*R47H/R47H*^ rats. This decrease is due to acute and reversible action of TNF-α and is not associated with increased human-Aβ levels and AD pathology. Thus, the p.R47H variant changes the excitatory/inhibitory balance, favoring excitation. This imbalance could potentiate glutamate excitotoxicity and contribute to neuronal dysfunction, enhanced neuronal death, and neurodegeneration. Future studies will determine whether this imbalance represents an early, Aβ-independent pathway leading to dementia and may reveal the AD-modifying therapeutic potential of TNF-α inhibition in the central nervous system.

Sporadic Alzheimer's disease (SAD) represents ∼95% of Alzheimer's disease (AD) cases. Yet, the most commonly used animal models are with familial AD (FAD) mutations, which only represent ∼5% of AD cases. This may be an issue because if FAD and SAD present significant pathogenic differences, therapeutic strategies effective in animals with FAD may have limited therapeutic efficacy in patients with SAD. Thus, model organisms that reproduce the pathogenesis of SAD would be helpful to identify therapeutic targets and test SAD-modifying therapeutics.

The p.R47H variant of the microglia gene triggering receptor expressed on myeloid cells 2 (*TREM2*) triples the risk of SAD in heterozygous carriers (1). Like other SAD-associated *TREM2* variants, the p.R47H variant impairs the amyloid β (Aβ)-clearing activities of microglia, presumably hampering elimination of toxic Aβ peptide forms (2, 3).

To study mechanisms by which this variant promotes SAD, we generated *Trem2*^*R47H*^ knock-in (KI) rats. These rats carry the R47H mutation in the rat *Trem2* gene and exhibit normal *Trem2* splicing and expression (4). *Trem2* is processed by A Disintegrin and Metalloproteinase 10; this cleavage releases a soluble N-terminal ectodomain (5, 6). Levels of soluble N-terminal ectodomain were not changed in the brains of young *Trem2*^*w/w*^, *Trem2*^*R47H/w*^, and *Trem2*^*R47H/R47H*^ rats (4). *Trem2*^*R47H*^ KI rats express two humanized *App* rat alleles that drive production of human Aβ (4). Hence, this KI model is useful to study both human Aβ–dependent and human Aβ–independent effects of the R47H mutation. In summary, *Trem2*^*R47H*^ KI rats represent a genetically faithful model organism of SAD.

Pathogenic mechanisms leading to SAD may start early in life. To reveal early dysfunctions that may lead, over time, to neurodegeneration, we have studied young *Trem2*^*R47H*^ KI rats. Preadolescent (4 weeks old) and periadolescent (6- to 8-weeks old) *Trem2*^*R47H*^ rats showed no alteration in steady-state levels of central nervous system (CNS) and cerebrospinal fluid (CSF) Aβ peptides (4, 7), suggesting that Aβ-clearance deficits caused by the p.R47H *TREM2* variant may manifest with aging. Yet, young *Trem2*^*R47H*^ rats present significant increased CNS and CSF concentrations of tumor necrosis factor-α (TNF-α), which cause augmented glutamatergic transmission and suppression of long-term potentiation (LTP) (7), an electrophysiological surrogate of learning and memory. Physiological levels of TNF-α produced by microglia are necessary to maintain normal surface expression of α-amino-3-hydroxy-5-methyl-4-isoxazole propionic acid (AMPA) receptors at postsynaptic termini; increased TNF-α concentrations promote rapid exocytosis of AMPA receptors in hippocampal pyramidal neurons, increasing the strength of glutamatergic synaptic responses (8, 9, 10, 11). The alterations in glutamatergic transmission found in *Trem2*^*R47H*^ rats are consistent with these effects of TNF-α and establish a direct link between a pathogenic variant of the microglia-specific *TREM2* gene and neuronal dysfunction of glutamatergic transmission and LTP.

In addition to boosting excitatory transmission, TNF-α decreases inhibitory synaptic strength by promoting endocytosis of γ-aminobutyric acid (GABA) receptors, hence reducing surface GABA receptors (10). Thus, in this study, we tested whether the p.R47H *TREM2* variant may reduce GABA transmission *via* increased brain TNF-α levels, with the purpose of determining whether the p.R47H *TREM2* variant changes the excitatory/inhibitory balance between glutamate and GABA transmission, favoring excitation. This unbalance could potentiate glutamate excitotoxicity and, over time, contribute to neuronal dysfunction, enhanced neuronal cell death, and neurodegeneration.

## Results

### Reduced inhibitory synaptic transmission at hippocampal SC–CA3>CA1 synapses of periadolescent rats carrying the *Trem2*^*R47H*^ variant

**Figure 1:**
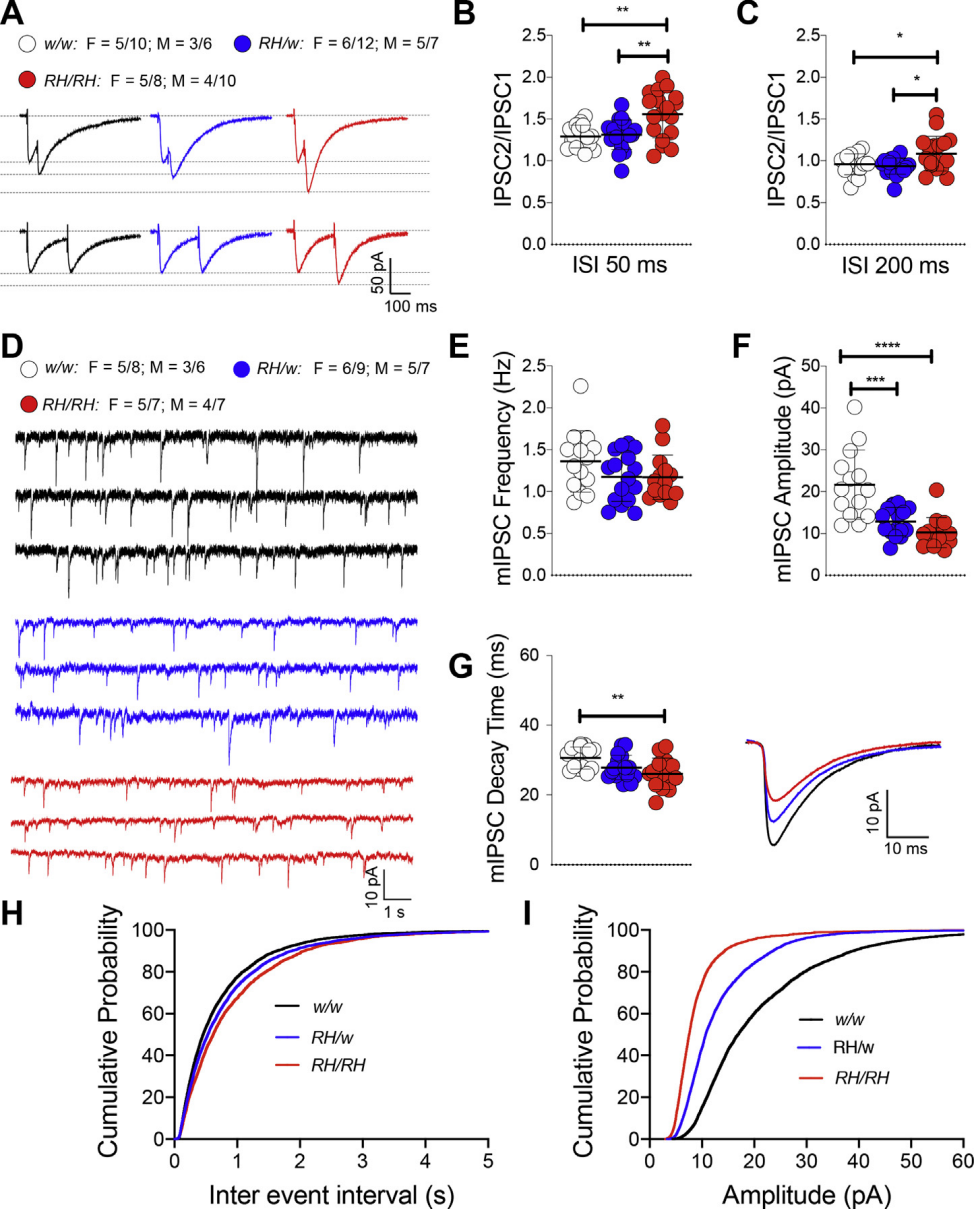
**Inhibitory GABAergic synaptic transmission is decreased in *Trem2***^***R47H***^**rats.***A*, representative traces of PPF of GABAergic transmission. *B*, plot of PPF at 50-ms ISI and the representative traces. [F (2, 50) = 8.968, *p* = 0.0005∗∗∗∗; post hoc Tukey's multiple comparisons test: *w/w* vs. *RH/w*, *p* = 0.9485 (ns); *w/w* vs. *RH/RH*, *p* = 0.0014∗∗; *RH/w* vs. *RH/RH*, *p* = 0.0022∗∗]. *C,* the plot of PPF at 200-ms ISI. [F (2, 50) = 5.106, *p* = 0.0096∗∗; post hoc Tukey's multiple comparisons test: *w/w* vs. *RH/w*, *p* = 0.9082 (ns); *w/w* vs. *RH/RH*, *p* = 0.0461∗; *RH/w* vs. *RH/RH*, *p* = 0.0118∗]. *D,* representative traces of mIPSCs. *E,* the plot of the frequency of mIPSCs. [F (2, 41) = 1.734, *p* = 0.1893(ns)]. Notably, *RH* mutant rats show mIPSCs with decreased frequency, albeit this decrease did not reach a statistical significance. *F,* the plot of the amplitude of mIPSCs. [F (2, 41) = 17.04, *p* < 0.0001∗∗∗∗; post hoc Tukey's multiple comparisons test: *w/w* vs. *RH/w*, *p* = 0.0002 ∗∗∗; *w/w* vs. *RH/RH*, *p* < 0.0001∗∗∗∗; *RH/w* vs. *RH/RH*, *p* = 0.3947(ns)]. *G,* the plot of the decay time of mIPSCs. [F (2, 41) = 5.254, *p* = 0.0093∗∗; post hoc Tukey's multiple comparisons test: *w/w* vs. *RH/w*, *p* = 0.1257(ns); *w/w* vs. *RH/RH*, *p* = 0.0070∗∗; *RH/w* vs. *RH/RH*, *p* = 0.3890 (ns)]. Representative averaged mIPSCs traces are shown on the *right*. Note that the amplitude and decay time of mIPSCs are significantly increased in RH mutant rats. *H,* the plot of the cumulative probability of mIPSCs interevent intervals. *I,* the plot of the cumulative probability of mIPSC amplitude. Data are represented as mean ± SD and were analyzed by ordinary one-way ANOVA followed by post hoc Tukey's multiple comparisons test when ANOVA showed significant differences. For each type of recordings, we indicate the number of animals by the genotype and sex, plus the number of recording by genotype and sex as follows: (1) genotypes: *w/w* = *Trem2*^*w/w*^, *RH/w* = *Trem2*^*R47H/w*^, *RH/RH* = *Trem2*^*R47H/R47H*^*;*(2) sex: F, female, M, males; (3) the number of animals and the number of recordings from animals: n/n’, where n = the number of animals and n’ = the number of recordings from the n animals. For example, the *w/w*: F = 5/10; M = 3/6 in panel A indicates that data for PPF for the *Trem2*^*w/w*^ rats were obtained from 5 female and 3 male rats and that 10 recordings were obtained from the 5 female and 6 recordings from the 3 male rats. GABA, γ-aminobutyric acid; ISI, inter stimulus interval; PPF, paired-pulse facilitation; mIPSCs, miniature inhibitory postsynaptic currents.

We examined the effects of the *Trem2*^*R47H*^ variant on GABAergic synaptic transmission in the hippocampal Schaffer-collateral pathway. First, we examined paired-pulse facilitation (PPF) of GABA_A_ receptor postsynaptic current, which is inversely correlated to the presynaptic GABA release probability. Our results show that PPF with 50- and 200-ms intervals is significantly increased in *Trem2*^*R47H/R47H*^ rats with SAD (Fig. 1, *A–C*), indicating GABA release is undermined in *Trem2*^*R47H/R47H*^ rats. Second, we analyzed miniature inhibitory postsynaptic currents (mIPSCs). The frequency of mIPSCs that largely reflects presynaptic GABA release probability is slightly decreased in both *Trem2*^*R47H/w*^ and *Trem2*^*R47H/R47H*^ rats; however, this decrease fails to achieve statistical significance (Fig. 1, *D*, *E* and *H*). Interestingly, the amplitude of mIPSCs that is dependent on levels of postsynaptic ionotropic GABA_A_ receptors was significantly decreased in both *Trem2*^*R47H/w*^ and *Trem2*^*R47H/R47H*^ rats (Fig. 1*D*, *F* and *I*), suggesting a reduction of GABA_A_ receptors on the postsynaptic surface. Moreover, we found that in *Trem2*^*R47H/R47H*^ rats, the decay time of mIPSC is shorter than in WT rats (Fig. 1*G*). As the subunit composition of the GABA_A_ receptor determines the decay time of mIPSCs (12), it is possible that the subunit composition of the GABA_A_ receptor is altered in *Trem2*^*R47H/R47H*^ rats. Altogether, these results suggest that the pathogenic variant p.R47H of the microglia gene *TREM2* leads to the reduction of GABAergic transmission to CA1 pyramidal neurons, and this effect is gene-dosage dependent.

### The reduced inhibitory synaptic transmission at hippocampal SC–CA3>CA1 synapses of periadolescent *Trem2*^*R47H*^ rats with SAD is caused by supraphysiological TNF-α

**Figure 2:**
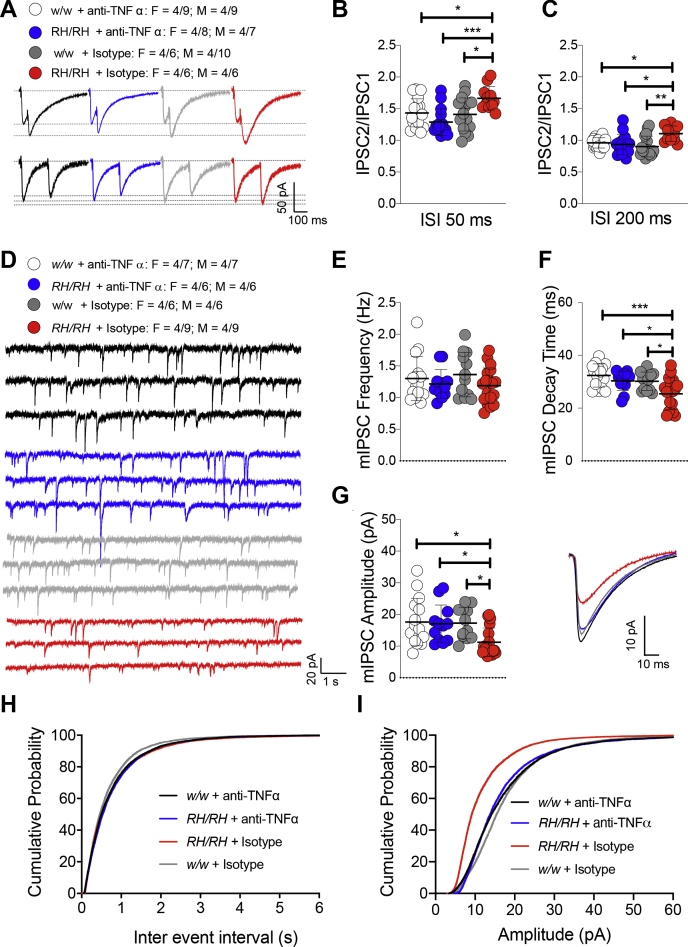
**The reduced inhibitory GABAergic synaptic transmission in *Trem2***^***R47H***^**rats is restored by reducing TNF-α function.***A,* representative traces of PPF of GABAergic transmission. Representative traces are averaged from 20 sweeps. *B,* the plot of PPF at 50-ms ISI. [F (3, 56) = 6.021, *p* = 0.0013∗∗; post hoc Tukey's multiple comparisons test: *w/w +* anti–TNF-α vs. *RH/RH +* anti–TNF-α, 0.2643 (ns); *w/w +* anti–TNF-α vs. *RH/RH +* isotype, 0.0452∗; *w/w +* anti–TNF-α vs. *w/w +* isotype, 0.9897 (ns); *RH/RH +* anti–TNF-α vs. *RH/RH +* isotype, *p* = 0.0005∗∗∗; *RH/RH +* anti–TNF-α vs. *w/w +* isotype *p* = 0.4452 (ns); *RH/RH +* isotype vs. *w/w +* isotype, *p* = 0.0267∗]. *C,* the plot of PPF at 200-ms ISI. [F (3, 56) = 5.361, *p* = 0.0026∗∗; post hoc Tukey's multiple comparisons test: *w/w +* anti–TNF-α vs. *RH/RH +* anti–TNF-α, 0.9485 (ns); *w/w +* anti–TNF-α vs. *RH/RH +* isotype, 0.0348∗; *w/w +* anti–TNF-α vs. *w/w +* isotype, 0.5884 (ns); *RH/RH +* anti–TNF-α vs. *RH/RH +* isotype, *p* = 0.0128∗; *RH/RH +* anti–TNF-α vs. *w/w +* isotype *p* = 0.9030 (ns); *RH/RH +* isotype vs. *w/w +* isotype, *p* = 0.0017∗∗]. Note that the increases in PPF of mIPSCs at 50 and 200 ms are reversed by anti–TNFα antibody application in RH mutant rats. *D,* representative traces of mIPSCs. *E,* the plot of the frequency of mIPSCs. [F (3, 53) = 0.9519, *p* = 0.4223 (ns)]. *F,* the plot of the amplitude of mIPSCs. [F (3, 53) = 4.562, *p* = 0.0065∗∗∗; post hoc Tukey's multiple comparisons test: *w/w +* anti–TNF-α vs. *RH/RH +* anti–TNF-α, 0.9945 (ns); *w/w +* anti–TNF-α vs. *RH/RH +* isotype, 0.0142∗; *w/w +* anti–TNF-α vs. *w/w +* isotype, 0.9989 (ns); *RH/RH +* anti–TNF-α vs. *RH/RH +* isotype, *p* = 0.0458∗; *RH/RH +* anti–TNF-α vs. *w/w +* isotype *p* = 0.9996 (ns); *RH/RH +* Isotype vs. *w/w +* isotype, *p* = 0.0350∗]. *G,* the plot of the decay time of mIPSCs. [F (3, 53) = 6.587, *p* = 0.0007∗∗∗; post hoc Tukey's multiple comparisons test: *w/w +* anti–TNF-α vs. *RH/RH +* anti–TNF-α, 0.6728 (ns); *w/w +* anti–TNF-α vs. *RH/RH +* isotype, *p* = 0.0005∗∗∗; *w/w +* anti–TNF-α vs. *w/w +* isotype, 0.6144 (ns); *RH/RH +* anti–TNF-α vs. *RH/RH +* isotype, *p* = 0.0342∗; *RH/RH +* anti–TNF-α vs. *w/w +* isotype *p* = 0.9997 (ns); *RH/RH +* isotype vs. *w/w +* isotype, *p* = 0.0436∗]. The representative averaged traces of the mIPSCs are shown on the *right*. Note that the decreased amplitude and decay time of mIPSCs in RH/RH rats was restored by anti–TNF-α antibody application. *H,* the plot of the cumulative probability of mIPSC interevent intervals. *I,* the plot of the cumulative probability of mIPSC amplitude. All data represent means ± SD. Data were analyzed by ordinary one-way ANOVA followed by post hoc Tukey's multiple comparison test when ANOVA showed significant differences. GABA, γ-aminobutyric acid; PPF, paired-pulse facilitation; mIPSCs, miniature inhibitory postsynaptic currents.

TNF-α produced by glia is necessary for physiological postsynaptic surface expression of both AMPA and GABA receptors. These two opposite effects of physiological TNF-α cooperate in maintaining physiological excitatory/inhibitory balance and the excitatory synaptic strength. Increased TNF-α concentration causes a swift surface AMPA receptor expression at postsynaptic termini and endocytosis of GABA receptors in hippocampal pyramidal neurons, changing the excitatory/inhibitory balance and favoring excitation (8, 9, 10, 11). Consistently, young *Trem2*^*R47H/R47H*^ rats show increased levels of TNF-α in the brain and CSF, which leads to enhanced glutamatergic transmission (7). To test whether TNF-α mediates the reduced inhibitory synaptic transmission at hippocampal SC–CA3>CA1 synapses of periadolescent rats carrying the *Trem2*^*R47H*^ variant, we treated hippocampal slices with a neutralizing antibody to rat TNF-α (anti–TNF-α), which functions as a TNF-α antagonist. To control for off-target effects of the antibody, we used a goat immunoglobulin (Ig)G isotype control. The 50% neutralization dose of this anti–TNF-α antibody against the cytotoxic effect of recombinant rat TNF-α (0.25 ng/ml) is about 500 ng/ml. Because physiological levels of TNF-α are necessary for normal glutamatergic transmission and most of the activities of TNF-α can be rapidly reversed (8, 9, 10, 11), we tested the acute effects of 10 ng/ml of anti–TNF-α, a concentration ∼50 times lower than the 50% neutralization dose. At this concentration, anti–TNF-α occluded the increased PPF (Fig. 2, *A–C*), the decreased mIPSC amplitude, and decay time (Fig. 2*D*, *F*, *G* and *I*) in *Trem2*^*R47H/R47H*^ rats. The goat IgG isotype control did not restore inhibitory GABAergic transmission alterations observed in the mutant rats (Fig. 2*D*, *F*, *G* and *I*) indicating that the effects of anti–TNF-α are specific. In addition, these low doses of anti–TNF-α do not alter inhibitory transmission in *Trem2*^*w/w*^ rats (Fig. 2, *A–I*), indicating that at least at this dosage, anti–TNF-α only targets GABAergic transmission alterations triggered by excess TNF-α set off by the *Trem2*^*R47H*^ variant. Overall, these data indicate that the decrease of GABAergic transmission at SC–CA3>CA1 synapses of *Trem2*^*R47H/R47H*^ rats is due to the acute action of supraphysiological TNF-α concentrations prompted by the *Trem2*^*R47H*^ variant and are rapidly reversible.

### Aβ peptides and oligomers levels are not changed in the brain of periadolescent *Trem2*^*R47H*^ rats

**Figure 3:**
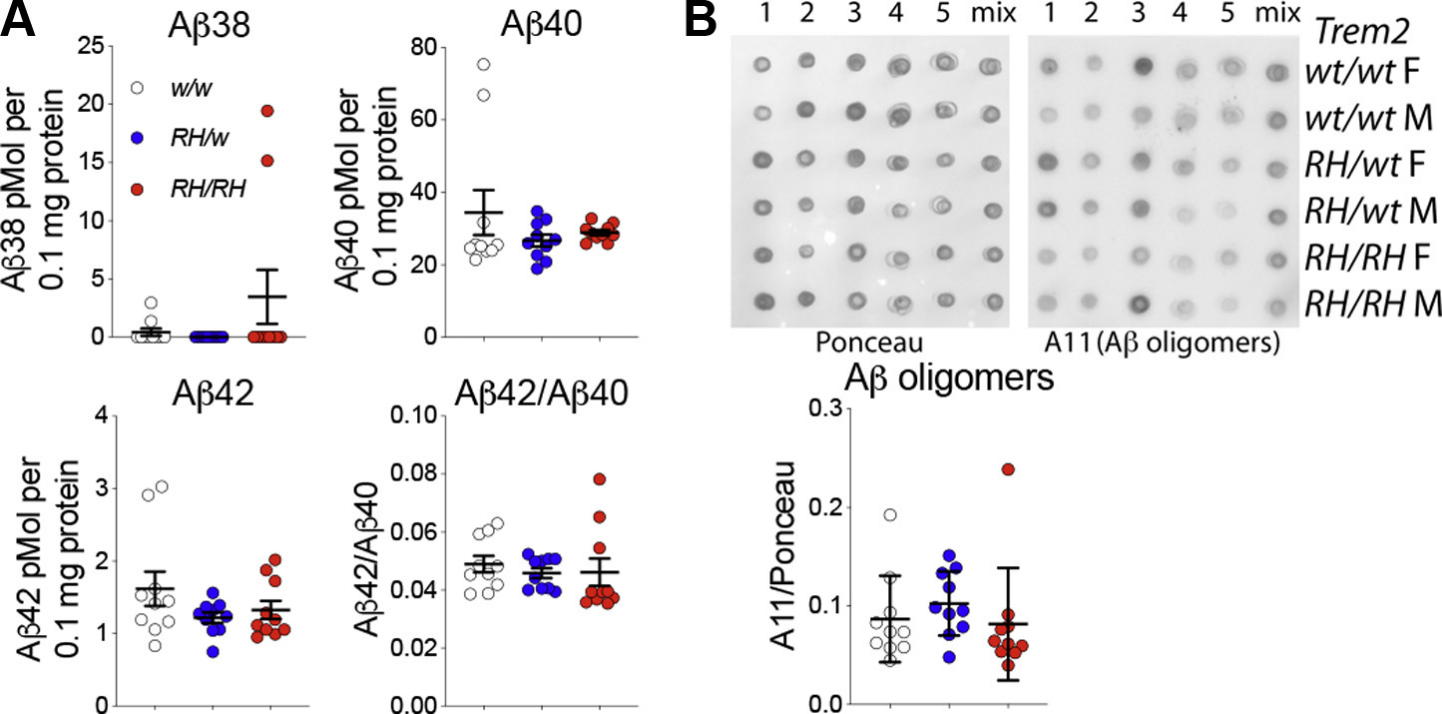
**Levels of human Aβ species are similar in the brain of periadolescent *Trem2***^***w/w***^**, *Trem2***^***R47H/w***^**, and *Trem2***^***R47H/R47H***^**rats.***A,* levels of Aβ38, Aβ40, and Aβ42/Aβ40 ratio in 7- to 8-week-old *Trem2*^*w/w*^, *Trem2*^*R47H/w*^, and *Trem2*^*R47H/R47H*^ rat brains. We used 5 male and 5 female rats for each genotype. Data are represented as mean ± SD. Data were analyzed by ordinary one-way ANOVA. No differences were seen in Aβ38 [F (2, 27) = 1.931, *p* = 0.1645], Aβ40 [F (2, 27) = 1.132, *p* = 0.3374], and Aβ42 [F (2, 27) = 1.668, *p* = 0.2074] levels and the Aβ42/Aβ40 ratio [F (2, 27) = 0.2683, *p* = 0.7667]. *B,* the same samples analyzed in panel A were used to determine levels of Aβ oligomers by dot blots using the oligomer-specific antibody A11. Before immunoblot analysis, membranes were stained with Ponceau red. Quantitative analysis of A11 blot was normalized to the Ponceau red quantitative analysis. The “mix” lane represents an equal mixture of all 5 samples of the same sex-genotype. Quantitation of the data is shown below the blots. Data are represented as mean ± SD. Data were analyzed by ordinary one-way ANOVA. No significant differences were seen among genotypes [F (2, 27) = 0.5667, *p* = 0.5740].

Analysis of brain homogenates from preadolescent rats (4) showed no significant alterations in levels of human Aβ40, Aβ42, and the Aβ42-to-Aβ40 ratio in *Trem2*^*R47H*^ rats, although the Trem2^R47H^ variant reduces binding and clearance of human Aβ *in vitro* (13). Previously, we found no changes in Aβ levels in the CSF of periadolescent animals as compared with *Trem2*^*w/w*^ rats (7). However, CSF concentrations of Aβ may not reflect the brain Aβ levels because aggregation of Aβ peptides in brain parenchyma may influence Aβ levels in the CSF. Thus, we assessed further Aβ levels in the brains of *Trem2*^*R47H*^ periadolescent animals, which were tested for glutamatergic (7) and GABAergic transmission (Figs. 1 and 2). No differences were seen in Aβ38, Aβ40, and Aβ42 levels and the Aβ42-to-Aβ40 ratio between periadolescent *Trem2*^*w/w*^, *Trem2*^*R47H/w*^, and *Trem2*^*R47H/R47H*^ rats (Fig. 3*A*), further suggesting that the reduced Aβ clearance caused by the Trem2^R47H^ variant *in vitro* does not result in significant alterations of Aβ steady-state levels *in vivo*, at least in preadolescent and periadolescent rats.

It has been postulated that toxic forms of Aβ are oligomers (14); we tested whether toxic oligomers are augmented in periadolescent *Trem2*^*R47H/w*^ and *Trem2*^*R47H/R47H*^ rats as compared with *Trem2*^*w/w*^ animals. To this end, we used the prefibrillar oligomer-specific antibody A11 to perform dot blots (15). We found no evidence supporting an increase in neurotoxic brain oligomer levels in periadolescent *Trem2* mutant rats with SAD as compared with WT rats (Fig. 3*B*).

### *Trem2*^*R47H/w*^ and *Trem2*^*R47H/R47H*^ adult (3-month-old) rat brains show no evidence of Aβ aggregation and neurodevelopmental or histopathological changes

**Figure 4:**
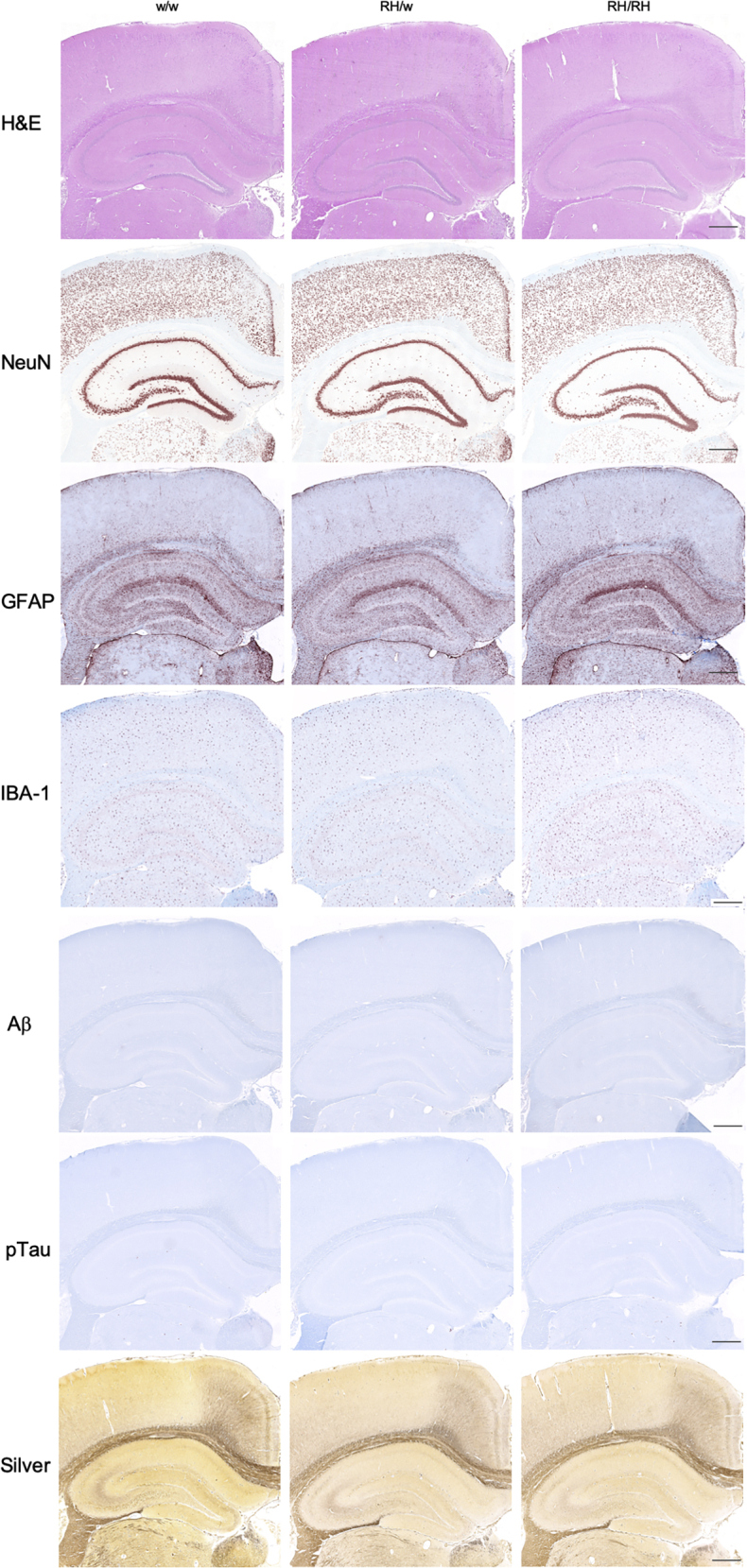
**Immunohistochemistry staining in 3-month-old *Trem2***^***w/w***^**, *Trem2***^***R47H/w***^**, and *Trem2***^***R47H/R47H***^**rats.** Representative images of the anterior hippocampus and overlaying somatosensory cortex of 3-month-old male *Trem2*^*w/w*^, *Trem2*^*R47H/w*^, and *Trem2*^*R47H/R47H*^ rat brains. Illustrates of, from the *top to bottom*, H&E, NeuN, GFAP, IBA-1, Aβ, pTau, and Bielschowski Silver staining, respectively. No observable differences in morphology (H&E) or neuronal (NeuN) cellularity are observed. The staining intensity of the microglial (IBA-1) and astrocytic (GFAP) markers are similar across all three genotypes. However, no Aβ or pTau expression can be observed, and no Bielschowski silver–stained plaques or tangles are present. Immunohistochemistry staining was performed on *Trem2*^*w/w*^ (4 male and 5 female rats), *Trem2*^*R47H/w*^ (4 male and 4 female rats), and *Trem2*^*R47H/R47H*^ (4 male and 4 female rats). The scale bar is equivalent to 500 microns. GFAP, glial fibrillary acidic protein.

**Figure 5:**
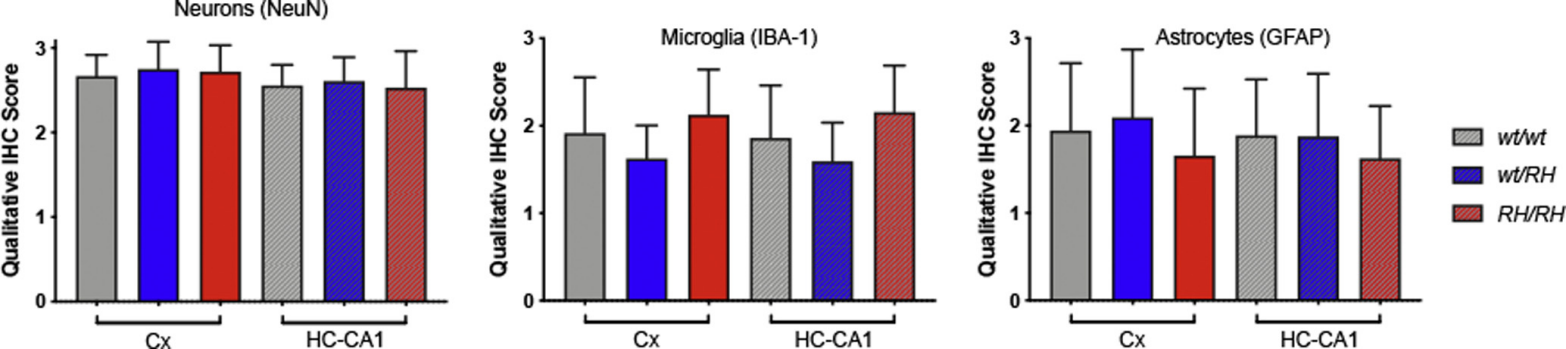
**Qualitative assessment of the NeuN, IBA-1, and GFAP staining in 3-month-old *Trem2***^***w/w***^**, *Trem2***^***R47H/w***^**, and *Trem2***^***R47H/R47H***^**rats.** Immunohistochemistry staining was scored in *Trem2*^*w/w*^ (4 male and 5 female rats), *Trem2*^*R47H/w*^ (4 male and 4 female rats), and *Trem2*^*R47H/R47H*^ (4 male and 4 female rats). Data are represented as mean ± SD of the qualitative score within the cortex and hippocampus-CA1 regions. Data were analyzed by ordinary one-way ANOVA within each brain region. No statistically significant differences were seen in NeuN_Cx_ [F (2, 21) = 1.736, *p* = 0.200], NeuN_HC-CA1_ [F (2, 21) = 0.533, *p* = 0.594], IBA-1_Cx_ [F (2, 22) = 0.375, *p* = 0.692], IBA-1_HC-CA1_ [F (2, 22) = 0.507, *p* = 0.609], GFAP_Cx_ [F (2, 22) = 0.159, *p* = 0.854], or GFAP_HC-CA1_ [F (2, 22) = 0.0237, *p* = 0.977]. GFAP, glial fibrillary acidic protein.

**Figure 6:**
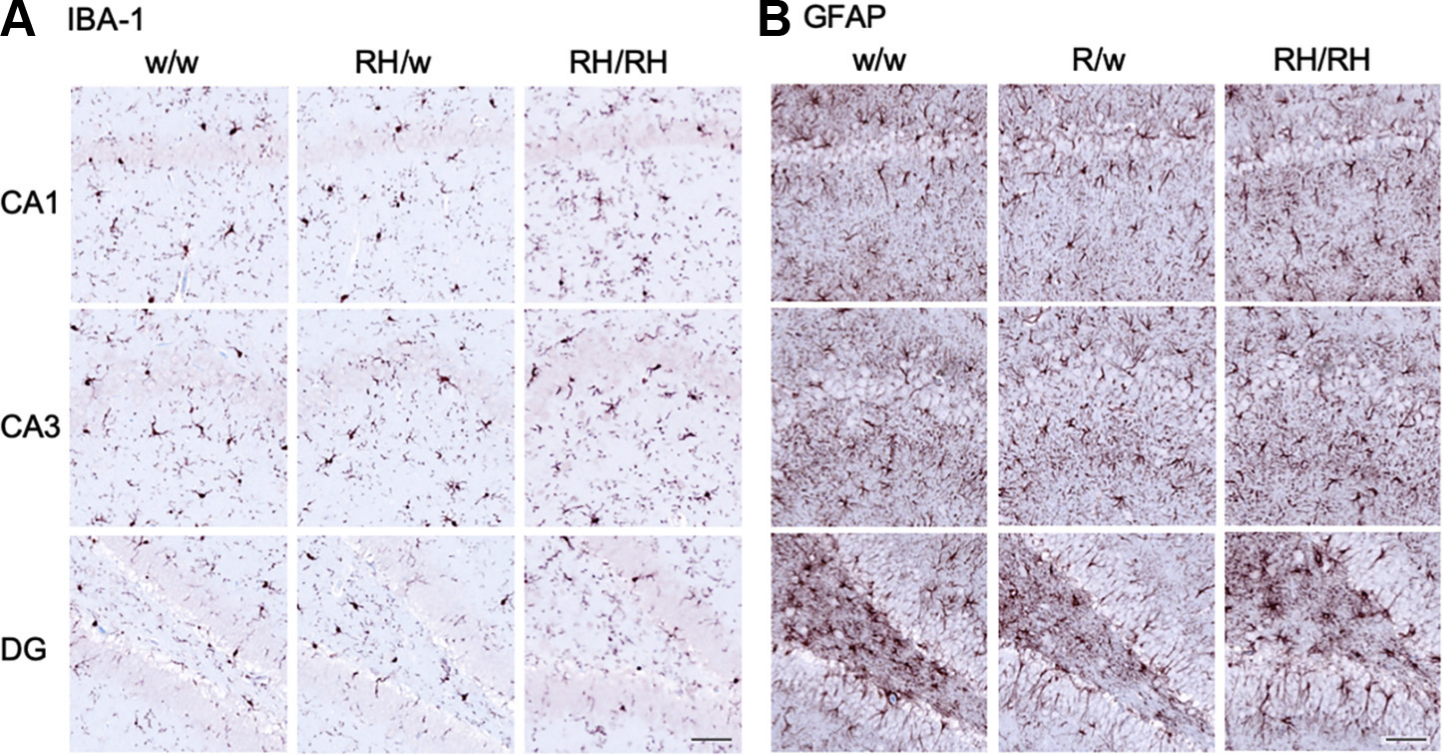
**Astrocyte and microglia immunohistochemistry staining in 3-month-old *Trem2***^***w/w***^**, *Trem2***^***R47H/w***^**, and *Trem2***^***R47H/R47H***^**rats.** Representative images illustrate microglia (*A***, IBA-1**) and astrocyte (*B***, GFAP**) staining with a *red-brown* chromagen in the dorsal hippocampus-CA1, hippocampus-CA3, and hippocampus-dentate gyrus (DG), of 3-month-old male *Trem2*^*w/w*^, *Trem2*^*R47H/w*^*,* and *Trem2*^*R47H/R47H*^ rat brains (*left to right*). The scale bar is equivalent to 500 microns. GFAP, glial fibrillary acidic protein.

The Aβ ELISA may not efficiently measure aggregated insoluble Aβ species. These species may trigger TNF-α release and impact neurodevelopment and/or cause overt neuropathology. To test these possibilities, we used histology and immunohistochemistry (IHC) to characterize brains from 3-month-old male and female *Trem2*^*w/w*^, *Trem2*^*R47H/w*^, and *Trem2*^*R47H/R47H*^ rats (see Table 1). We tested 3-month-old rats to increase the possibility of detecting pathology that may start in preadolescent rats, which may be detectable by histology and IHC only weeks later. Regions of analysis included the frontal cortex, cingulate cortex, whole hippocampus, and entorhinal cortex. No gross morphological changes were evident by H&E staining in any of the rats analyzed (Fig. 4). Qualitative inspection of NeuN staining showed no appreciable changes in the neuronal density in any of the regions analyzed. Qualitative analysis performed on the hippocampus (CA1) and the somatosensory cortex did not indicate overt neuronal loss in *Trem2*^*R47H/w*^ and *Trem2*^*R47H/R47H*^ rats as compared with *Trem2*^*w/w*^ rats (Fig. 5). Despite the presence of elevated proinflammatory cytokines in the brain and CSF of *Trem2*^*R47H/R47H*^ rats and to a lesser extent of *Trem2*^*R47H/w*^ rats, no evidence of significant astrocytosis or microgliosis was observed (Figure 4, Figure 5, Figure 6), a evaluated by the staining intensity (Fig. 5) and cellular morphology of glial fibrillary acidic protein and ionized calcium-binding adapter molecule 1 (IBA1)-stained tissues (Fig. 6, *A*–*B*). Although a trend toward higher IBA-1 staining intensity was observed in the Trem2^R47H/R47H^ rats than in Trem2^w/w^ and *Trem2*^*R47H/w*^ rats, this was not statistically significant. The microglia presented numerous fine processes, characteristic of the resting state, and did not present obvious intermediate or amoeboid morphologies with enlarged processes or cell bodies (Fig. 6*A*) in all three genotypes. Similarly, astrocytes did not present hypertrophy of soma and processes in any of the groups (Fig. 6*B*). Amyloid plaques, as measured by simultaneous costaining with the anti-Aβ antibodies 6E10 and 4G8, were absent in all tissues analyzed (Fig. 4). Those results are consistent with the similar Aβ40 or Aβ42 levels in *Trem2*^*w/w*^, *Trem2*^*R47H/w*^, and *Trem2*^*R47H/R47H*^ rats as well as the absence of plaque in 3-month-old rats with humanized Aβ (16). Moreover, Tau phosphorylation as measured by AT8 immunostaining was absent in all the groups (Fig. 4), and a modified Bielschowsky silver staining did not reveal plaques, dystrophic neurites, or axonal pathologies in any of the tissues analyzed (Fig. 4). Overall, histological analysis of these rats shows no obvious evidence of neurodevelopmental impairments, neurodegeneration, neuroinflammation, or AD-like pathology at 3 months of age.

**Table 1:** Primary and amplification antibodies used for IHC

Target	Antigen antibody/clone (supplier)	Antigen retrieval	Dilution	Secondary and amplification
Neurons	NeuN, Mouse monoclonal A60 (Millipore)	Citrate, pH 6.0 HIER	1:3000	RbαM and GtαRb-HRP
Amyloid beta	1–16 and 17–24 beta amyloid, Mouse monoclonal 6E10, and 4G8 (Biolegend)	80% Formic acid	1:1000	RbαM and GtαRb-HRP
Microglia	IBA-1, Rabbit polyclonal (Wako)	Citrate, pH 6.0 HIER	1:2000	DkαRb-bio and SA-HRP
Phospho-Tau	Phospho-Tau (Ser202, Thr205), mouse monoclonal AT8 (ThermoFisher)	Citrate, pH 6.0 HIER + 10-min PK	1:1000	RbαM and GtαRb-HRP
Astrocytes	GFAP, Rabbit polyclonal (Thermo Scientific)	Citrate, pH 6.0 HIER	1:200	DkαRb-bio and SA-HRP

GFAP, glial fibrillary acidic protein; IHC, immunohistochemistry; HIER, heat-induced antigen retrieval; PK, proteinase K; M, mouse; Rb, rabbit; Gt, goat; Dk, donkey; bio, biotin; SA, streptavidin; HRP, horseradish peroxidase.

## Discussion

Proinflammatory cytokines, especially TNF-α, are significantly increased in the brain and CSF of young *Trem2*^*R47H*^ rats (7)*.* Consistent with the evidence that (1) TNF-α produced by glia controls postsynaptic physiological expression of AMPA receptors and (2) increased concentrations of TNF-α cause rapid AMPA receptor exocytosis increasing excitatory synaptic strength (8, 9, 10, 11), we found that supraphysiological TNF-α concentrations boost glutamatergic transmission and suppress LTP, a surrogate of learning and memory, in periadolescent *Trem2*^*R47H*^ rats with SAD (7)*.*

**Figure 7:**
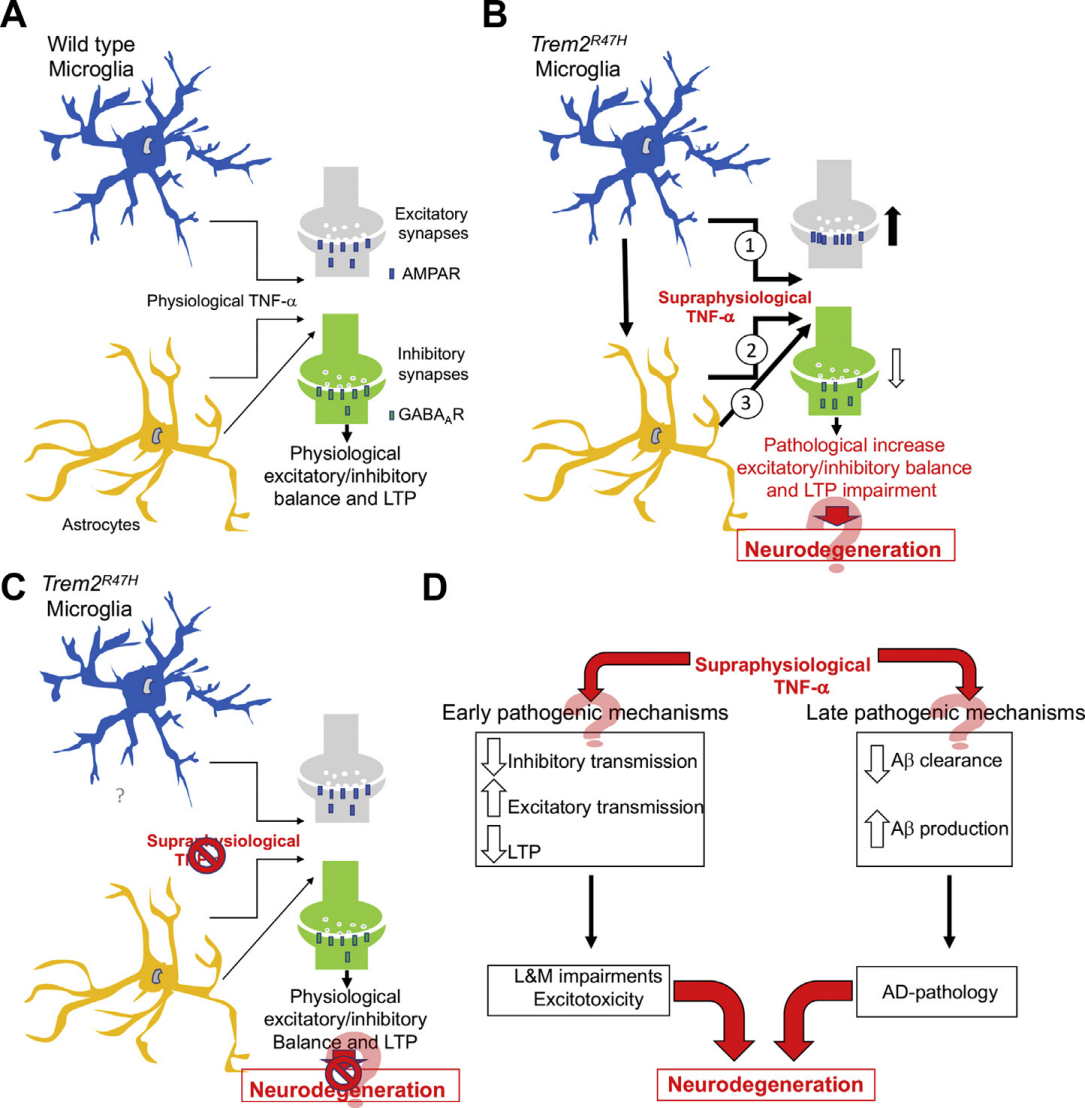
**Model depicting how the p.R47H TREM2 variant may enhance the excitatory/inhibitory balance and cause neurodegeneration.***A,* astrocytes and microglia set physiological excitatory/inhibitory balance and LTP *via* TNF-α. *B, TREM2* expression is restricted to microglia: thus, it is likely that microglia expressing the p.R47H variant are the source of supraphysiological TNF-α 1. *Trem2*^*R47H*^ microglia may also promote TNF-α production by other cell types, such as astrocytes 2. Finally, TNF-α produced by microglia may modulate the synaptic regulatory functions of astrocytes 3. These possibilities are not mutually exclusive. Supraphysiological TNF-α impairs LTP and increases the excitatory/inhibitory balance by enhancing glutamate transmission and reducing GABA transmission. A swift surface exocytosis of AMPA receptors and endocytosis of GABA receptors at postsynaptic termini could be one mechanism by which supraphysiological TNF-α increases the excitatory/inhibitory balance and reduces LTP. These early dysfunctions may enhance glutamate excitotoxicity and neuronal cell death, culminating into obvert cognitive deficits, brain pathology, and neurodegeneration decades later. *C,* resetting TNF-α activity at physiological levels normalizes excitatory/inhibitory balance and LTP and could prevent neurodegeneration. *D,* supraphysiological TNF-α may promote neurodegeneration *via* early and late pathogenic mechanisms. Early pathogenic mechanisms would include interfering with synaptic transmission; late pathogenic mechanisms would include progressing amyloid pathology by favoring Aβ production over clearance. GABA, γ-aminobutyric acid; LTP, long-term potentiation.

TNF-α also physiologically regulates, in an opposite manner, inhibitory synaptic strength by promoting endocytosis GABA receptors, hence reducing surface GABA receptors at inhibitory synapses (10). In accord, we show here that young *Trem2*^*R47H*^ rats have reduced GABA responses (Fig. 1). Low doses of a neutralizing anti–TNF-α antibody occlude these alterations (Fig. 2) indicating that supraphysiological TNF-α concentrations impair GABAergic transmission in *Trem2*^*R47H*^ rats. This evidence also indicates that GABAergic deficits must be due to an acute and constant action of supraphysiological TNF-α. TNF-α–dependent synaptic transmission alterations occur in the absence of changes in steady-state levels of soluble Aβ (Fig. 3 and (4, 7)) and obvious evidence of neurodevelopmental impairments, neurodegeneration, neuroinflammation, or AD-like pathology (Figure 4, Figure 5, Figure 6). Overall, these data suggest that the TNF-α–dependent synaptic transmission alterations caused by the p.R47H *TREM2* variant are independent of, and perhaps precede, changes in Aβ steady-state levels and brain pathology. TNF-α binds 2 receptors: TNFR1 is ubiquitously expressed, whereas TNFR2 is mainly expressed by immune and endothelial cells (17). Future studies will be needed to address the contribution of TNFR1 and TNFR2 to this synaptic dysfunction of TNF-α.

Several data support the hypothesis that pathogenic *TREM2* variants hamper the toxic Aβ-clearing activities of microglia (2, 3) and that elevated TNF-α levels cause increased Aβ production (18, 19, 20, 21) and reduced microglial clearance of Aβ (22), yet we see no evidence of altered Aβ metabolism in young *Trem2*^*R47H*^ rats. A possible explanation for this apparent discrepancy is that the effects of the p.R47H *TREM2* variant and of supraphysiological TNF-α levels become evident in an aging-dependent manner, whereas alterations in synaptic transmission and LTP are early dysfunctional events. A longitudinal analysis of Aβ metabolism and AD-related amyloid pathology in aging *Trem2*^*R47H*^ rats will address this question. Because *TREM2* expression in the brain is restricted to microglia (23), it is plausible that *Trem2*^*R47H*^ microglia may be the source of supraphysiological TNF-α. *Trem2*^*R47H*^ microglia may also promote TNF-α production by other cell types such as astrocytes. The possibilities that non–brain-resident cells may be the source of extra TNF-α or that TNF-α clearance is altered in *Trem2*^*R47H*^ rats cannot be discounted. In addition, TNF-α is a strong activator of astrocytes, which release a range of regulators of synaptic transmission. Thus, TNF-α may regulate synaptic function by modulating the synaptic regulatory functions of astrocytes, which are closely associated with synapses (24). These possibilities do not need to be mutually exclusive

Interestingly, although these observations may suggest that increased levels of TNF-α and other cytokines measured in *Trem2*^*R47H/R47H*^ are attributable to microglia or astrocytes, their immunoactivity does not appear to be associated with obvious morphological changes. However, we cannot exclude a mildly activated microglia phenotype, characterized by increased branching of thin processes as well as lengthening of processes and the secretion of proinflammatory cytokines. Studies using a model of prion disease (25) have indicated that microglia can switch to a phenotype contributing to neuronal damage without morphological changes (25). Thus, in some experimental models of CNS disease, there is no direct correlation between the morphological profile and functional phenotype in microglia.

SAD is a disease of old age. Therefore, it is reasonable to assume that changes in the excitatory/inhibitory balance between glutamate (7) and GABA transmission (this article), favoring excitation, caused by the p.R47H *TREM2* variant have no relevance to SAD pathogenesis. However, it is possible that these early dysfunctions may potentiate glutamate excitotoxicity enhancing neuronal cell death and culminate into obvert cognitive deficits, brain pathology, and neurodegeneration decades later. In this context, it is worth mentioning that several genes linked to dementia, including *APP*, *PSEN1*, *PSEN2,* and *ITM2b*, play a physiological role in glutamatergic transmission and that mutations linked to familial dementia alter this physiological functions (16, 26, 27, 28, 29, 30, 31, 32, 33, 34, 35, 36, 37, 38, 39, 40, 41).

Many observations have linked TNF-α and its receptors to dementia pathogenesis. TNF-α levels are significantly elevated in the CSF and CNS of patients with AD (42, 43, 44, 45, 46); in addition, *TNF-α*, *TNFR1,* and *TNFR2* gene polymorphisms are associated with SAD (47, 48). Transgenic mouse models of FAD show elevated TNF-α levels, which cause increased Aβ production (18, 19, 20, 21) and reduced microglial clearance of Aβ (22). Ablation of *TNFR1* reduced Aβ formation, Aβ plaques, and cognitive deficits (49). However, ablation of both TNFR1 and TNFR2 exacerbated Aβ and tau pathology because of aggravation of TNFR1-mediated FAD pathology resulting from silencing of TNFR2 (50).

The most potent TNF-α–specific inhibitors (TNFIs) approved by the food and drug administration are biologic drugs, which are used for treatment of peripheral inflammatory conditions, including rheumatoid arthritis, Crohn disease, and psoriasis. Biologic TNFIs include recombinant fusion proteins (etanercept, an ∼125-kDa fusion protein, consisting of a TNFR2 domain coupled to the Fc portion of human IgG_1_ (51)) and anti–TNF-α monoclonal antibodies (adalimumab, infliximab, golimumab, and certolizumab (52)). Preclinical studies have demonstrated the protective effects of TNFI, when administered by intracerebroventricular injection, on Aβ pathology, tau phosphorylation, and cognitive deficits (53, 54, 55, 56). Remarkably, perispinal etanercept administration and intrathecal administration of infliximab resulted in cognitive improvements in patients with AD (57, 58). This human and model organism evidence has suggested a role of TNF-α in neurodegeneration. Here, we show that the pathogenic p.R47H *TREM2* variant changes the excitatory/inhibitory balance favoring excitation and that these changes happen early in life, are dependent on supraphysiological TNF-α, and are independent of changes in Aβ steady-state levels and brain pathology (Fig. 7, *A–C*).

In summary, supraphysiological TNF-α might trigger dementia *via* early and late pathogenic mechanisms (Fig. 7*D*): 1) early mechanisms include altering the excitatory/inhibitory balance favoring excitotoxic neuronal cell death, impairing LTP and learning and memory, and (2) late mechanisms include increasing brain pathology by favoring Aβ production over Aβ clearance. On the whole, these data underscore the AD-modifying therapeutic potential of TNFIs in the CNS.

## Experimental procedures

### Rats and ethics statement

All experiments were performed according to policies on the care and use of laboratory animals of the Ethical Guidelines for Treatment of Laboratory Animals of the National Institutes of Health. The procedures were described and approved by the Rutgers Institutional Animal Care and Use Committee (protocol number 201702513). All efforts were made to minimize animal suffering and reduce the number of animals used. The animals were housed two per cage under controlled laboratory conditions with a 12-h dark–light cycle and a temperature of 22 ± 2°C. Rats had free access to standard rodent diet and tap water. The background strain of the rat model is Long-Evans.

### Brain slice preparation

Rats aged 6 to 8 weeks were deeply anesthetized with isoflurane and then intracardially perfused with 20-ml ice-cold cutting solution containing (in mM) 120 choline chloride, 2.6 KCl, 26 NaHCO_3_, 1.25 NaH_2_PO_4_, 0.5 CaCl_2_, 7 MgCl_2_, 1.3 ascorbic acid, and 15 glucose. The brains were rapidly removed from the skull. Coronal brain slices containing the hippocampal formation (400 μm thick) were prepared in the ice-cold cutting solution bubbled with 95% O_2_/5% CO_2_ using Vibratome VT1200S (Leica Microsystems, Germany). The slices were transferred into an interface chamber in artificial cerebrospinal fluid (ACSF) containing (in mM) 126 NaCl, 3 KCl, 1.2 NaH_2_PO_4_; 1.3 MgCl_2_, 2.4 CaCl_2_, 26 NaHCO_3_, and 10 glucose (at pH 7.3), bubbled with 95% O_2_ and 5% CO_2_, and incubated at 30 °C for 1 h and then kept at room temperature afterward. The hemislices were transferred to a recording chamber perfused with ACSF at a flow rate of ∼2 ml/min using a peristaltic pump. Experiments were performed at 28.0 ± 0.1 °C.

### Electrophysiological recording

Whole-cell recordings in the voltage-clamp mode (-70 mV) were made with patch pipettes containing (in mM) 135 KCl, 2 MgCl_2_, 0.1 EGTA, 10 HEPES, 2 Na_2_ATP, 0.2 Na_2_GTP, and 5 QX-314 (pH 7.3, osmolarity 290–310 mOsm). Patch pipettes (resistance, 8–10 MΩ) were pulled from 1.5-mm thin-walled borosilicate glass (Sutter Instruments, Novato, CA) on a horizontal puller (model P-97; Sutter Instruments, Novato, CA).

CA1 neurons were viewed under upright microscopy (FN-1, Nikon Instruments, Melville, NY) and recorded with an Axopatch-700B amplifier (Molecular Devices, San Jose, CA). Data were low-pass–filtered at 2 kHz and acquired at 5 to 10 kHz. The series resistance (Rs) was consistently monitored during recording in case of reseal of ruptured membrane. Cells with Rs >20 MΩ or with Rs deviated by >20% from initial values were excluded from analysis. Basal postsynaptic synaptic responses were evoked at 0.05 Hz by electrically stimulating the Schaffer collateral afferents using concentric bipolar electrodes. Inhibitory postsynaptic currents (IPSCs) were recorded with membrane potential held at -70 mV in ACSF containing 10-μM 2,3-dioxo-6-nitro-7-sulfamoyl-benzo[f]quinoxaline to block AMPA receptor current. The stimulation intensity was adjusted to evoke IPSCs that were 40% of the maximal evoked amplitudes (“test intensity”). For recording of paired-pulse ratio, paired-pulse stimuli with 50-ms or 200-ms interpulse interval were given at test intensity. The paired-pulse ratio was calculated as the ratio of the second IPSC amplitude to the first. mIPSCs were recorded by maintaining neurons at -70 mV with ACSF containing 1-μM TTX to block action potentials and 1-μM 2,3-dioxo-6-nitro-7-sulfamoyl-benzo[f]quinoxaline to block AMPA receptor current. mIPSCs were recorded for 5 to 10 min for analysis. Data were collected and analyzed using the Axopatch 700B amplifiers and pCLAMP10 software (Molecular Devices), and mIPSCs are analyzed using Mini Analysis Program.

### Antibody treatment

Right after slice-cutting, 10 ng/ml goat anti–TNF-α (AF-510-NA, R&D Systems) or goat IgG control (AB-108-C, R&D Systems) was incubated and perfused throughout recordings. Experiments were performed at 28.0 ± 0.1 °C.

### Rat brain preparation for ELISA

Rats were anesthetized with isoflurane and perfused *via* intracardiac catheterization with ice-cold PBS. This perfusion step eliminates cytokines and Aβ present in the blood. Brains were extracted and homogenized using a glass-teflon homogenizer (w/v = 100-mg tissue/1-ml buffer) in 250-mM sucrose, 20-mM Tris-base, pH 7.4, 1-mM EDTA, 1-mM EGTA plus protease and phosphatase inhibitors (ThermoScientific), with all steps carried out on ice or at 4 °C. Total lysate was solubilized with 0.1% SDS and 1% NP-40 for 30-min rotating. Solubilized lysate was spun at 20,000g for 10 min, and the supernatant was collected and analyzed by ELISA.

### ELISA

Aβ38, Aβ40, and Aβ42 were measured with V-PLEX Plus Aβ Peptide Panel 1 6E10 (K15200G) and V-PLEX Plus Aβ Peptide Panel 1. Measurements were performed according to the manufacturer’s recommendations. Plates were read on a MESO QuickPlex SQ 120. Data were analyzed using Prism software and represented as mean ± SD.

### Dot-blot analysis

The same samples used for ELISA were tested for Aβ oligomers by dot blot. Briefly, 2.5 μg of material was spotted with a p20 pipette on a nitrocellulose membrane. After the membranes were dried, proteins were visualized by Ponceau red staining. Membranes were blocked for 30 min in 5% milk (Bio-Rad 1706404) and washed extensively in PBS/Tween20 0.05%, and a primary antibody (rabbit polyclonal antibody A11 raised against oligomeric Aβ) was applied overnight at 4°C, at 1:1000 dilution in the blocking solution (Thermo 37573). A 1:1 mix of horseradish peroxidase–conjugated anti-rabbit (Southern Biotech, OB405005) and anti-rabbit (Cell Signaling, 7074) was diluted 1:1000 in 5% milk and used against A11 antibody for 30 min at room temperature, with shaking. Blot was developed with West Dura ECL reagent (Thermo, PI34076) and visualized on a ChemiDoc MP Imaging System (Bio-Rad). Signal intensity was quantified with Image Lab software (Bio-Rad). Data were analyzed using Prism software and represented as mean ± SD.

### Tissue preparation and staining

Rat brain tissue was fixed and stored in 70% ethanol after transcardiac perfusion with PBS and 4% paraformaldehyde fixative. All tissues were dehydrated through graded ethanol and xylene, infiltrated with paraffin wax, and embedded in paraffin blocks. Slides were manually deparaffinized and rehydrated before the automated IHC. Slides initially underwent antigen retrieval, by one of the following methods, heat-induced epitope-retrieval (HIER), HIER and proteinase K treatment, or formic acid treatment. HIER was performed by incubation in a citrate buffer (pH 6.0) and heating to 100 °C for a period of 60 min. When performed before the 10-min proteinase K treatment, citrate HIER was limited to 20 min. Formic acid treatment was a 10-min incubation in 80% formic acid, followed by washing in tris-buffered saline-Tween 20. All IHC studies were performed at room temperature on a Lab Vision Autostainer. Briefly, slides were incubated sequentially with hydrogen peroxide for 5 min, to quench endogenous peroxidase, followed by 5 min in a protein block, and then incubated with primary antibodies as outlined in Table 1. Antibody binding was amplified using the appropriate secondary reagents (20 min), followed by a horseradish peroxidase conjugate (20 min), and visualized using the aminoethyl carbazole chromogen (20 min). All IHC sections were counterstained with Acid Blue 129 and mounted with an aqueous mounting medium (59).

### Statistical analysis

Data were analyzed using GraphPad Prism software and expressed as mean ± SD. Statistical tests used to evaluate significance are shown in figure legends. Significant differences were accepted at *p* < 0.05.

## Data availability

The datasets used and/or analyzed during the present study are available from the corresponding author on reasonable request.

## Conflict of interest

The authors declare that they have no conflicts of interest with the contents of this article.
